# Lung Function-Based Assessment of Small Airway Dysfunction in Chronic Respiratory Diseases

**DOI:** 10.3390/biology15191765

**Published:** 2026-10-04

**Authors:** Chalerm Liwsrisakun, Athavudh Deesomchok, Chaicharn Pothirat, Warawut Chaiwong

**Affiliations:** Division of Pulmonary, Critical Care and Allergy, Department of Internal Medicine, Faculty of Medicine, Chiang Mai University, Chiang Mai 50200, Thailand; chalerm.liw@cmu.ac.th (C.L.); athavudh.d@cmu.ac.th (A.D.); chaicharn.p@cmu.ac.th (C.P.)

**Keywords:** small airways, chronic respiratory diseases, asthma, COPD, interstitial lung diseases, airway remodeling

## Abstract

Small airway dysfunction (SAD) is increasingly recognized as an early and clinically important component of chronic respiratory diseases. Because pathological changes in the peripheral airways may remain clinically silent until disease is advanced, sensitive methods to detect and characterize SAD are required to improve phenotyping and support mechanism-based therapeutic development. This narrative review summarized key SAD mechanisms, including epithelial injury and dysregulated repair, mucus retention, inflammation-driven mucosal edema, smooth muscle dysfunction, and matrix remodeling, that contribute to small-airway narrowing and ventilation heterogeneity. We synthesized lung function approaches used to quantify SAD and relate measurement outputs to functional abnormalities, including ventilation heterogeneity, small-airway narrowing, airway closure, and gas trapping. We reviewed commonly used clinical and research assessments, including spirometry, forced oscillation techniques (FOTs), impulse oscillometry (IOS), multiple-breath washout (MBW), fractional exhaled nitric oxide (FeNO), and static lung volume measurements. We also highlighted the practical advantages, key limitations, and interpretive pitfalls of each modality and proposed a pragmatic framework for integrating these physiological tests into clinical evaluation and phenotypic identification of SAD. Finally, we identified priorities for standardization and future studies to determine how lung function-based SAD endpoints can guide treatment decisions and serve as outcome measures in clinical trials.

## Simple Summary

Small airway dysfunction (SAD) is a common and clinically relevant feature of chronic respiratory diseases, including asthma and COPD. It may occur early and can be present despite normal or near-normal spirometry. While spirometry, with bronchodilator testing when appropriate, remains the first-line assessment, oscillometry offers a practical office-based method to assess peripheral airway mechanics and airway closure, particularly in asthma with preserved spirometry. Static lung volumes and multiple-breath washout can provide complementary information on gas trapping and ventilation heterogeneity, mainly in specialized settings. However, interpretation of oscillometry should consider device-specific reference values, measurement quality, and clinical context because universal cut-points for SAD have not yet been established.

## 1. Introduction

Small airway dysfunction (SAD) is increasingly recognized as a key and frequently early pathophysiological feature across a spectrum of chronic respiratory diseases, including asthma, chronic obstructive pulmonary disease (COPD), and bronchiolitis. Moreover, SAD appears clinically relevant in several additional conditions in which distal airway pathology contributes to symptom burden, physiological impairment, and disease progression, including selected interstitial lung diseases (ILDs) with concomitant small-airway involvement [1]. The “small airways”, typically defined as conducting airways with an internal diameter < 2 mm, comprise an extensive branching network in which relatively modest alterations in luminal caliber, wall thickness, mucus burden, or airway–parenchymal tethering can produce clinically meaningful disturbances in ventilation distribution and expiratory lung emptying [2].

Historically, pathology of the peripheral airways was regarded as clinically “silent”, largely because conventional spirometric indices may remain within normal limits until disease becomes extensive or abnormalities propagate to more proximal airways [3]. However, a growing body of mechanistic and clinical evidence indicates that SAD meaningfully affects key outcomes, including symptom burden, particularly exertional dyspnea, exacerbation risk, responsiveness to inhaled therapies, and the emergence of gas trapping and dynamic hyperinflation [4]. Accordingly, there is a compelling rationale to develop and standardize sensitive physiological approaches capable of detecting and characterizing SAD, facilitating phenotypic stratification, and providing robust endpoints for mechanism-based therapeutic interventions [5].

This narrative review synthesizes the following: (i) the mechanistic basis of SAD, with emphasis on epithelial injury and disordered repair, mucus retention, inflammation-related mucosal oedema, smooth-muscle dysfunction, and extracellular matrix remodeling; (ii) lung function methodologies used to quantify SAD; (iii) the extent to which specific test outputs reflect physiological abnormalities, including ventilation heterogeneity, small-airway narrowing, airway closure, and gas trapping; and (iv) the principal practical strengths, limitations, and interpretive pitfalls of each technique. This narrative review addressed these gaps by synthesizing lung function-based SAD assessment across multiple modalities and major chronic respiratory diseases, including a focused appraisal of the recent literature on oscillometry and small-airway assessment, and emphasizing interpretive frameworks, strengths/limitations, and clinically relevant use-cases. We subsequently proposed a pragmatic approach for integrating these assessments across clinical and research settings and highlight priority directions for future investigation.

## 2. Methods

This article was conducted as a narrative review focusing on lung function-based approaches for assessing SAD in chronic respiratory diseases. To inform the review, we performed a structured literature search in PubMed/MEDLINE, Embase, Scopus, and Web of Science. Searches covered publications from database inception through 31 August 2026, with the main search conducted between December 2025 and August 2026.

The search strategy combined controlled vocabulary terms, where available, with free-text terms related to small airways, physiological assessment, and chronic respiratory diseases. Core search concepts included the following: “small airway dysfunction”, “small airways disease”, “small airway disease”, “oscillometry”, “impulse oscillometry”, “forced oscillation technique”, “multiple-breath washout”, “multiple breath washout”, “ventilation heterogeneity”, “spirometry”, “FEF25–75”, “mid-expiratory flow”, “lung volume measurement”, “plethysmography”, “closing volume”, “inert gas washout”, and “fractional exhaled nitric oxide” or “FeNO”. These terms were combined with disease-related terms, including “asthma”; “chronic obstructive pulmonary disease” or “COPD”; “bronchiolitis”; “bronchiectasis”; “cystic fibrosis”; and “interstitial lung disease” or “ILD”. A representative PubMed search strategy was as follows: (“small airway dysfunction” OR “small airways disease” OR “small airway disease” OR “small airways”) AND (“oscillometry” OR “impulse oscillometry” OR “forced oscillation technique” OR “multiple breath washout” OR “ventilation heterogeneity” OR “spirometry” OR “FEF25–75” OR “mid-expiratory flow” OR “plethysmography” OR “lung volumes” OR “closing volume” OR “inert gas washout” OR “FeNO” OR “fractional exhaled nitric oxide”) AND (“asthma” OR “COPD” OR “chronic obstructive pulmonary disease” OR “bronchiolitis” OR “bronchiectasis” OR “cystic fibrosis” OR “interstitial lung disease” OR “ILD”).

Reference lists of key reviews, consensus statements, guidelines, and highly relevant primary studies were also examined to identify additional publications. Only English-language articles were included. Eligible publications included original research studies, clinical trials, observational studies, methodological studies, consensus documents, and selected narrative or systematic reviews that reported lung function-derived indices intended to reflect SAD or distal airway involvement in chronic respiratory diseases. Studies were prioritized when they (1) evaluated a physiological measure relevant to small-airway or distal-airway function; (2) included clinically relevant respiratory disease populations; (3) examined associations with symptoms, disease control, exacerbations, imaging findings, or treatment response; (4) provided information on measurement performance, repeatability, interpretability, or proposed thresholds; or (5) represented influential guidelines, consensus recommendations, or landmark studies. Studies focused exclusively on acute respiratory infection, non-chronic respiratory conditions, animal models, or physiological measures without relevance to SAD assessment were excluded. Titles and abstracts were reviewed for relevance, followed by full-text assessment of potentially informative publications. Because this was a narrative review rather than a systematic review, formal duplicate screening, a standardized risk-of-bias assessment, and meta-analysis were not undertaken. When multiple studies addressed the same physiological modality or clinical question, evidence was synthesized qualitatively, with greater weight given to studies with robust methodology, larger and well-characterized populations, longitudinal or interventional designs, external validation, and consistency with the broader literature. For studies proposing different diagnostic thresholds, we emphasized the variability introduced by device type, population characteristics, reference equations, and outcome definitions, rather than endorsing a single universal cut-off when validation was insufficient.

Evidence was organized thematically according to lung function modality, including spirometry, body plethysmography and static lung volumes, oscillometry, washout techniques, FeNO, and other physiological assessments. The aim was to provide a clinically oriented overview of the strengths, limitations, interpretation, and potential role of each modality in identifying SAD across chronic respiratory disease phenotypes.

## 3. Conceptualizing SAD: From Pathology to Functional Abnormalities

### 3.1. Defining SAD

In this review, it is important to distinguish (i) anatomical small-airway pathology from (ii) physiological abnormalities that are compatible with small-airway involvement but are not, by themselves, specific.

#### 3.1.1. Anatomical Small-Airway Pathology

“Small airways” typically refer to the distal conducting airways (classically <2 mm in internal diameter). Pathology in these airways includes wall thickening and remodeling, inflammation and edema, mucus impaction, smooth muscle hypertrophy/bronchoconstriction, luminal narrowing, and in more advanced disease, airway loss/obliteration. These abnormalities are directly observed with histopathology and, in some settings, inferred from imaging; however, they are not directly measured by routine lung function tests [6,7].

#### 3.1.2. Physiological SAD

By contrast, in physiological terms, SAD refers to a pattern of measurable functional disturbance that is consistent with dysfunction of distal conducting airways, arising from heterogeneous narrowing, closure, or obliteration and from altered airway–parenchymal interactions [6]. Importantly, no single lung function test uniquely identifies anatomical small-airway disease, because similar physiological abnormalities can also be produced (or strongly influenced) by factors such as altered elastic recoil, heterogeneity of ventilation from multiple causes, or changes in operating lung volumes [7,8].

Accordingly, throughout this review, SAD is conceptualized as a probabilistic physiological construct: the confidence that “small airways are the dominant site of abnormality” increases when multiple complementary physiological signatures converge, particularly those obtained during tidal breathing (e.g., forced oscillation techniques and washout tests) rather than exclusively during forced expiration [7,9,10,11].

### 3.2. Physiological Signatures Associated with SAD (Not Individually Specific)

From a lung function standpoint, physiological SAD is typically inferred from one or more downstream functional consequences. Each is compatible with small-airway pathology, but none is fully specific when considered in isolation.

#### 3.2.1. Increased Peripheral Airway Resistance/Small-Airway Narrowing

Small-airway narrowing may be suggested by disproportionate impairment of expiratory flow at lower lung volumes, frequency-dependent increases in airway resistance, and/or abnormal respiratory impedance during tidal breathing [7]. While these findings are consistent with distal airway involvement, they may also be affected by heterogeneous bronchoconstriction, changes in lung volume, and upstream airway behavior, underscoring the need for integrated interpretation [7].

#### 3.2.2. Heightened Airway Closure Propensity

A tendency toward expiratory airway closure and volume-dependent airflow limitation may manifest as more negative or otherwise abnormal reactance and physiological evidence compatible with early airway closure and development of poorly ventilated or non-ventilated lung units [8]. However, closure-related physiological patterns may also be influenced by parenchymal mechanics and airway–parenchymal tethering, so they should be interpreted as markers of dysfunctional peripheral mechanics rather than as direct proof of a single anatomical lesion [7,8].

#### 3.2.3. Gas Trapping and Hyperinflation

Gas trapping and hyperinflation are reflected by elevated residual volume (RV) and RV/total lung capacity (TLC), increased functional residual capacity (FRC), and/or reduced inspiratory capacity (IC). Dynamic hyperinflation during exertion adds clinically meaningful information, particularly for exertional dyspnea [12]. Nevertheless, gas trapping is not specific to small-airway narrowing alone; it can also reflect altered elastic recoil and airway collapse mechanisms that interact with peripheral airway disease [7,12].

#### 3.2.4. Ventilation Heterogeneity

Ventilation heterogeneity arises from regional variation in time constants due to patchy obstruction and/or closure and can be quantified using washout-derived indices, such as the lung clearance index (LCI) [9]. While such indices are often sensitive to distal airway abnormalities, ventilation heterogeneity can also emerge from mixed mechanisms across airway generations, therefore functioning best as a sensitive indicator of abnormal ventilation distribution, not a uniquely localizing test [9].

Integrative concept (how to interpret “SAD” in practice).

These processes often coexist and amplify one another: mucus retention and inflammation-associated mucosal edema can narrow distal airways and increase time-constant heterogeneity; delayed emptying predisposes to gas trapping and, under physiological stress, dynamic hyperinflation [12,13,14,15,16,17]. For this reason, the conceptual framework used here is that physiological SAD is best assessed by combining tests that probe different consequences of peripheral airway dysfunction, particularly those reflecting resistance/impedance during tidal breathing and ventilation distribution, alongside measures of trapping/hyperinflation [7,9,10,11].

### 3.3. Why “Normal Spirometry” Does Not Exclude Clinically Important Physiological SAD

Conventional spirometry measures the integrated behavior of the respiratory system during forced expiration and is influenced substantially by larger-airway caliber, expiratory effort, and global mechanical properties [18]. Small-airway pathology, by contrast, is frequently heterogeneous and patchy; if a sufficient proportion of lung units remains relatively well ventilated, global spirometric indices, especially forced expiratory volume in the first second (FEV_1_), may remain within or near the normal range despite clinically relevant abnormalities in ventilation distribution and persistent symptoms [19,20].

Consistent with this, among individuals with asthma and normal spirometry, SAD was identified in 38.6% when defined by impulse oscillometry (IOS), compared with 7.7% in healthy subjects using the same IOS-based definition [21]. Moreover, forced expiratory maneuvers may transiently recruit partially closed airways or alter airway caliber compared with quiet tidal breathing, reducing sensitivity to abnormalities most prominent at resting lung volumes [10,11]. These limitations support a complementary strategy using tidal-breathing physiological tools, notably FOT/IOS and MBW, to detect functional abnormalities compatible with small-airway involvement when spirometry is normal or only mildly abnormal [9,10,11].

## 4. Mechanisms of Small Airway Dysfunction

### 4.1. Epithelial Injury and Dysregulated Repair

The small-airway epithelium is a barrier interface governing immune surveillance and mucociliary clearance [22]. In asthma and COPD, recurrent injury (allergens, pollutants, viral infections, cigarette smoke, biomass exposure) can compromise epithelial integrity and trigger maladaptive repair programs [22,23]. When repair is dysregulated, epithelial remodeling, goblet cell metaplasia, and airway-wall structural changes may reduce luminal caliber and alter airway mechanics [22,24], shaping physiological signatures that are compatible with SAD (e.g., abnormal impedance and increased ventilation heterogeneity), sometimes even when spirometric impairment is modest [5,25].

### 4.2. Mucus Retention and Impaired Mucociliary Clearance

Mucus plugging is common in asthma, COPD (notably chronic bronchitis phenotypes), bronchiolitis, and bronchiectasis, often with heterogeneous distribution [13,14]. In distal airways, small mucus aggregates can markedly reduce cross-sectional area, promoting low-volume airflow limitation and regions of effectively absent ventilation, particularly when accompanied by mucosal edema [15,16]. Physiologically, this pattern is often associated with increased ventilation heterogeneity (washout abnormalities), increased closure propensity, and gas trapping (elevated RV and RV/TLC) [26,27], with oscillometric changes consistent with peripheral airflow limitation and closure-related abnormalities in resistance/reactance metrics [26,28].

### 4.3. Inflammation-Driven Mucosal Edema

Inflammatory mediator release increases microvascular permeability, causing mucosal edema that narrows the lumen and increases wall thickness [29,30]. This mechanism is relevant across eosinophilic asthma, neutrophilic COPD, and mixed patterns in other chronic airway disorders [31,32,33]. Because inflammation varies over time and can modify physiology and treatment responsiveness [34,35], edema may contribute to fluctuating airflow limitation and potentially reversible components of physiological SAD [4,5], observable as changes in bronchodilator response patterns, oscillometry-derived resistance/reactance, and washout indices tracking disease activity [36,37,38,39].

### 4.4. Smooth Muscle Dysfunction

Airway smooth muscle (ASM) determines baseline tone and bronchoconstrictor capacity [40]. In asthma, ASM hypertrophy/hyperplasia and altered signaling can produce exaggerated narrowing, particularly in distal airways where small baseline radii magnify resistance changes [41,42]. In COPD, ASM remodeling may coexist with reduced elastic recoil, weakening airway–parenchymal tethering and promoting expiratory narrowing/collapse [32,43]. Physiologically, this may present as bronchial hyperresponsiveness and partially reversible narrowing [44], with effects captured not only by FEV_1_ but also by indices of air trapping (e.g., forced vital capacity (FVC) and/or RV changes) and oscillometry-derived measures, sometimes with symptomatic benefit disproportionate to spirometric change [45,46].

### 4.5. Matrix Remodeling and Loss of Airway–Parenchymal Interdependence

Extracellular matrix remodeling (including fibrosis and altered wall composition) can thicken/stiffen small airways, while emphysematous destruction reduces elastic recoil and airway–parenchymal tethering [7,47]. These changes promote fixed narrowing and increased expiratory collapse tendency. This is prominent in COPD and bronchiolitis and can also occur in long-standing asthma [48,49]. In selected ILD, peribronchiolar fibrosis or inflammatory bronchiolitis may contribute to mixed physiology and/or ventilation heterogeneity disproportionate to spirometric impairment [50,51]. Functionally, matrix remodeling is associated with persistent gas trapping, reduced low-volume expiratory flow, and reactance abnormalities, often with limited reversibility to bronchodilator therapy alone [52,53].

**Figure 1:**
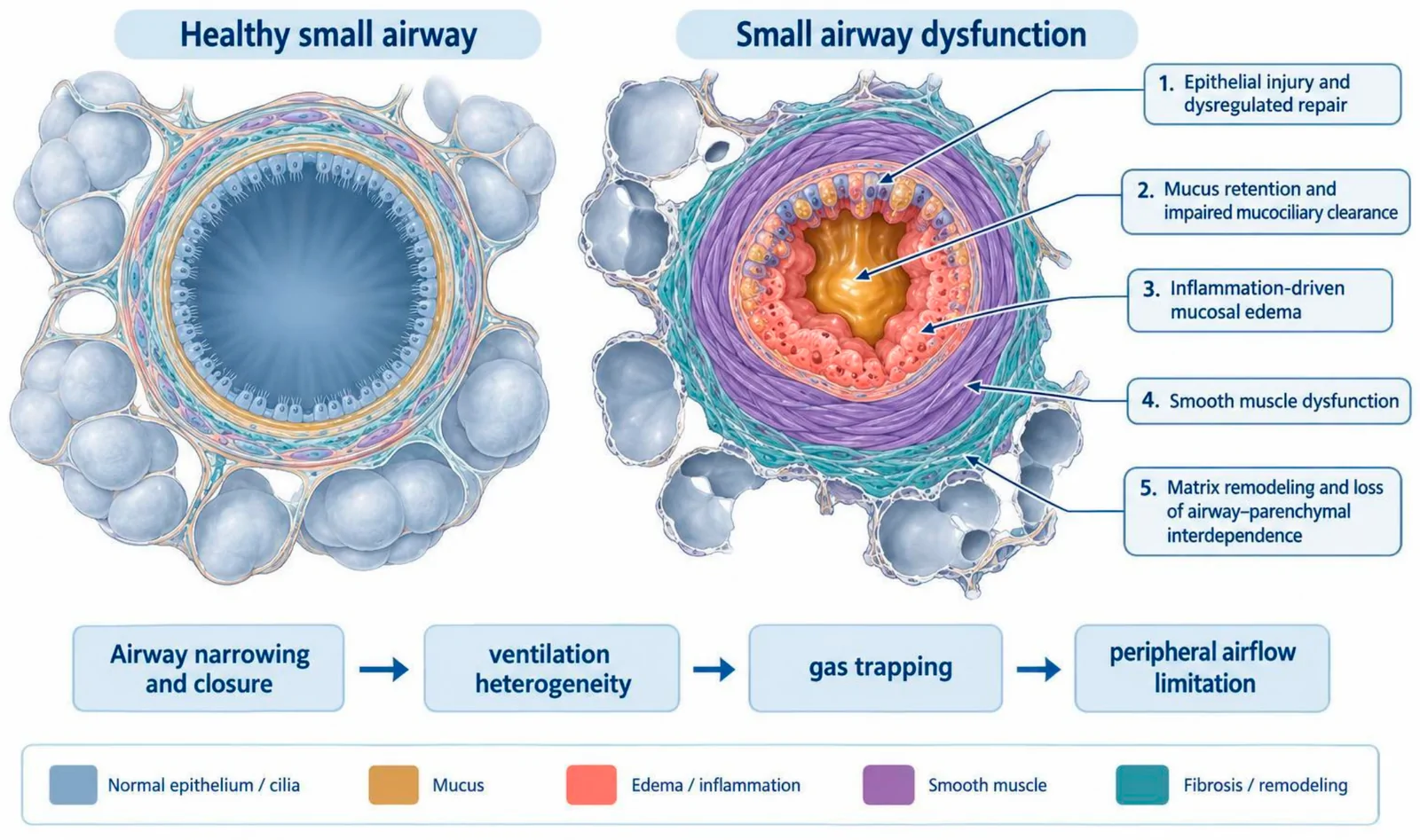
Schematic overview of the mechanisms underlying SAD.

To facilitate understanding of the structural components of SAD, Figure 1 provides a representative schematic illustration of the major pathological mechanisms, including epithelial injury and dysregulated repair, mucus retention, inflammation-driven mucosal edema, smooth muscle dysfunction, and matrix remodeling with loss of airway–parenchymal interdependence.

## 5. Lung Function Approaches to Quantify SAD: Linking Outputs to Functional Abnormalities

No single physiological test provides a comprehensive characterization of SAD. Available modalities interrogate distinct domains of respiratory physiology, including expiratory flows, static and dynamic lung volumes, respiratory impedance, ventilation distribution, and, in selected contexts, markers of airway inflammation. A pragmatic interpretive strategy is therefore to integrate results according to four fundamental functional abnormalities that commonly underpin SAD: ventilation heterogeneity, small-airway narrowing (increased peripheral resistance), airway closure (increased closing tendency), and gas trapping/hyperinflation.

### 5.1. Spirometry

#### 5.1.1. Conventional Indices (FEV_1_, FVC, FEV_1_/FVC)

Spirometry remains a cornerstone of respiratory assessment, forming the basis for the diagnosis of airflow obstruction and the grading of its severity in many clinical algorithms [54]. Nevertheless, FEV_1_ represents an integrated outcome determined by airway resistance, lung elastic recoil, and the patient’s expiratory effort, rather than a selective measure of distal airway function [55]. Although obstructive spirometric patterns often imply small-airway involvement, spirometry alone has limited capacity to localize pathology to the peripheral airways or to disentangle distal contributions from more proximal abnormalities [56].

In practice, spirometry serves primarily as an indirect indicator of airway narrowing and, in more advanced disease, may show associations with gas trapping and hyperinflation [57]. However, it can be relatively insensitive to early-stage or spatially heterogeneous SAD and may underestimate abnormalities that are most evident during quiet tidal breathing rather than forced maneuvers [58]. Notably, some work suggests that spirometry-derived indices beyond the conventional triad may enhance sensitivity to mild distal obstruction. In particular, the ratio (forced expiratory volume in the third second (FEV_3_)-FEV_1_)/FVC has been proposed as a simple, low-cost marker that may improve the detection and prediction of SAD in asthma, especially among patients with otherwise preserved spirometry, by capturing subtle airflow limitation and airway hyperresponsiveness that may not be evident in standard spirometric indices alone [59].

#### 5.1.2. Mid-Expiratory Flows—Forced Expiratory Flow Between 25% and 75% of the FVC (FEF25–75), Forced Expiratory Flow at 50% of the FVC (FEF50), and Forced Expiratory Flow at 75% of the FVC (FEF75)

Mid- to late-expiratory flow indices, such as FEF25–75, FEF50, and FEF75, are frequently cited as supportive markers of peripheral airflow limitation [60]. In particular, a reduced FEF25–75 in the presence of a preserved FEV_1_/FVC ratio has, in some settings, been interpreted as suggestive of “early” small-airway impairment [61]. In adults with asthma, reduced FEF25–75% may be associated with a greater burden of physiological abnormalities compatible with SAD. Even in the presence of normal FEV_1_, lower FEF25–75% has been associated with airway hyperresponsiveness, eosinophilic airway inflammation, and greater disease severity [62]. More recently, evidence from a large cohort has indicated that % predicted FEF25–75 may align more closely with COPD airway pathology and associated physiological impairment, independent of % predicted FEV_1_ [63]. However, lower FEF25–75% values identified a comparatively smaller subset of patients and showed generally weak relationships with physiological indices of peripheral airway resistance. These findings suggest that reductions in FEF25–75% may not consistently reflect small-airway involvement, particularly in milder asthma [64].

In the study by Liwsrisakun et al., reduced FEF25–75% was used as a spirometric definition of SAD. Using the study-specific threshold of <60% predicted, reduced FEF25–75% was observed in a high proportion of participants with COPD (95.5%) and asthma with fixed airflow obstruction (FAO) (98.2%), but less frequently in those with asthma without FAO (19.8%) and healthy controls (2.6%) [21]. However, mid-expiratory flows have important limitations. They display considerable intra- and inter-individual variability and are strongly influenced by FVC and expiratory time; as a consequence, low values may reflect a reduced FVC due to true restriction, gas trapping, or suboptimal technique/effort rather than isolated distal airway dysfunction [65]. In addition, clinical cut-points are inconsistently standardized, and specificity for distal airway pathology is limited [66]. Indeed, Quanjer and colleagues have argued that FEF25–75 does not provide incremental value for routine clinical decision-making beyond conventional spirometric indices [65]. Accordingly, FEF indices are best regarded as adjunctive signals that warrant corroboration with complementary evidence—such as oscillometry, static lung volumes, washout measures, symptom and exacerbation profiles, and (where available) imaging—rather than as stand-alone markers of SAD [67,68,69,70].

#### 5.1.3. Flow-Volume Curve Morphology

Morphological features of the spirometric flow-volume loop, particularly concavity of the descending expiratory limb, may reflect heterogeneous airway emptying and distal airway obstruction [71,72,73]. In a population-based spirometry dataset, quantitative measures of central and peripheral concavity of the expiratory curve identified substantially more individuals with features consistent with early small-airway obstruction (approximately twice as many as would be detected using FEF25–75%) and demonstrated greater specificity for respiratory symptoms. These findings suggest that concavity metrics may add clinically informative small-airway signals to routine spirometric assessment [72]. Complementing this, another study reported that an objective spirometric metric capturing late-expiratory concavity/flattening can identify a relatively small subgroup with abnormal values, most of whom had conditions commonly associated with small-airway pathology. Importantly, the previous work implies that abnormal concavity in the context of otherwise normal spirometric ratios should prompt consideration of further evaluation for small-airway disease, particularly in symptomatic individuals with “normal spirometry” by conventional criteria [73]. Although quantitative concavity indices have been described, they are not consistently available or implemented across pulmonary function laboratories. When such indices are available and demonstrate adequate reproducibility, expiratory-limb concavity can serve as supportive evidence for suspected SAD, especially when interpreted alongside symptoms and corroborating physiological measures [72].

### 5.2. Static Lung Volumes and Gas Trapping (Plethysmography and Gas Dilution)

Measurement of static lung volumes provides direct physiological insight into gas trapping and hyperinflation, abnormalities that commonly arise from distal airway closure and/or prolonged regional emptying [74]. Among these indices, RV and the RV/TLC ratio are frequently interpreted as markers of gas trapping [74,75]. FRC may be increased in the setting of hyperinflation; in particular, plethysmographic FRC often rises with airflow obstruction and air trapping [76]. A reduced IC is another clinically important signal of hyperinflation and has been linked to dyspnea and exercise limitation, especially in COPD [76,77]. Collectively, these metrics most directly quantify the gas trapping/hyperinflation domain of SAD and, more indirectly, reflect airway closure and heterogeneous lung emptying [74].

The RV/TLC ratio is often especially informative because TLC may be elevated in obstructive lung disease, such that RV expressed relative to TLC can better reflect the proportion of trapped gas. In asthma, Sorkness et al. reported higher RV/TLC in severe compared with non-severe disease, with an inverse relationship to FVC, consistent with a gas-trapping contribution to reduced vital capacity [78]. In COPD, resting hyperinflation defined by lung volumes (RV/TLC ≥ 40%) was independently associated with increased all-cause mortality in a cohort of 353 patients [79]. The European Respiratory Society (ERS) report proposed pragmatic cutoffs for identifying air-trapping consistent with small-airway involvement, defining an abnormal RV/TLC as >126% predicted (or >48% absolute) by plethysmography and >128% predicted (or >47% absolute) by helium dilution [80]. When SAD was defined by an RV/TLC threshold of ≥40%, diagnoses of asthma with airflow obstruction (odds ratio: 6.35; 95% CI: 2.43, 16.58) and COPD (odds ratio: 2.98; 95% CI: 1.05, 8.45) were markedly associated with SAD, supporting RV/TLC ≥ 40% as a clinically relevant marker of air-trapping–related small-airway involvement [81].

Interpretation, however, requires appropriate reference standards. Applying a fixed threshold (e.g., defining RV or RV/TLC as abnormal at 120% predicted) may overestimate gas trapping because the upper limit of normal (ULN) for RV varies substantially with age, height, and sex; values approximating 140–150% predicted may still fall within the ULN in younger individuals, whereas the ULN is lower in older adults (approximately 125–135% predicted). Accordingly, abnormality should be defined using appropriate ULN- and predicted-based reference equations rather than a single universal cut-point [82].

Methodological considerations are also crucial. Gas dilution techniques (e.g., helium dilution) can underestimate lung volumes when poorly ventilated or non-communicating regions fail to equilibrate with the tracer gas, whereas body plethysmography measures compressible intrathoracic gas and may therefore capture non-communicating volumes [74,83]. Consequently, divergence between dilution and plethysmographic volumes can itself provide indirect evidence of trapped gas and marked ventilation heterogeneity [84]. Conversely, in severe obstruction, plethysmography may overestimate thoracic gas volume if panting frequency and technique are suboptimal, underscoring the importance of rigorous protocol adherence and quality control when comparing methods or tracking change over time [74,83,85].

### 5.3. Forced Oscillation Techniques/Impulse Oscillometry (FOT/IOS)

Oscillometry (FOT and IOS) quantifies respiratory system impedance during quiet tidal breathing, thereby providing effort-independent information with particular sensitivity to abnormalities arising in the peripheral airways [86]. In principle, small pressure oscillations are applied across a range of frequencies, and the resulting pressure-flow relationship is analyzed to derive impedance and its components [87]. Resistance (Rrs) represents the resistive load of the respiratory system. Low-frequency resistance (e.g., R5) reflects contributions from both central and peripheral airways, whereas higher-frequency resistance (e.g., R20) is weighted more strongly toward the central airways [87]. The frequency dependence of resistance commonly expressed as R5-R20 is frequently interpreted as a marker of ventilatory heterogeneity and peripheral airway involvement [86,87]. Reactance (Xrs) reflects the combined elastic and inertive properties of the respiratory system; more negative low-frequency reactance is generally consistent with reduced effective compliance and/or an increased tendency toward airway closure and ventilation heterogeneity [86,87]. Derived summary metrics, such as the area of reactance (AX) and resonant frequency (Fres), are also associated with peripheral airway mechanics and closing propensity [86,87]. Taken together, oscillometry is particularly well suited to infer (i) small-airway narrowing via frequency-dependent resistance and (ii) airway closure/ventilation heterogeneity via reactance abnormalities [87,88]. A major practical advantage is that IOS can be a sensitive and relatively specific reflection of small-airway mechanics while requiring only tidal breathing rather than maximal forced expiratory effort [86]. Evidence supporting clinical relevance includes population- and disease-specific observations. For example, a large community-based cohort used R5-R20 as an index of peripheral resistance to classify IOS-defined SAD. IOS-defined SAD was associated with poorer lung function, higher airway resistance, and greater CT evidence of emphysema and gas trapping than in individuals without SAD, supporting the ability of oscillometry to detect early “quiet-zone” abnormalities [89]. In adults with asthma and preserved spirometry, IOS, particularly R5-R20, has also been reported to discriminate uncontrolled asthma more effectively than spirometry [36]. IOS also showed potential clinical utility for identifying physiological abnormalities compatible with SAD in individuals with chronic respiratory symptoms [90]. In symptomatic individuals with preserved pulmonary function, increases in IOS-derived resistance and reactance measures were associated with SAD, supporting IOS as a symptom-correlated and sensitive adjunct to spirometry for the early detection of SAD [91].

Clinically, oscillometry can reveal abnormalities when spirometry is normal or near-normal and is especially useful in individuals unable to perform reliable forced maneuvers (e.g., children, older adults, or those with severe disease) [92]. In asymptomatic smokers with preserved spirometry, IOS has detected early mechanical dysfunction—e.g., higher R5 and R5-R20, increased AX/Fres, and more abnormal reactance, such as lower X5—with reported associations to smoking exposure and reduced exercise capacity, supporting a role for IOS in identifying small-airway impairment prior to overt spirometric decline [93]. Oscillometry is also attractive for monitoring bronchodilator responsiveness and therapeutic effects that primarily alter distal airway mechanics [94]. Important limitations relate to technical and interpretive standardization. Results may vary by device, signal processing, and protocol, such that reference equations and cut-points are not necessarily interchangeable across manufacturers or laboratories [95,96,97]. Artefacts due to upper-airway shunting, inadequate cheek support, or glottic behaviors can distort impedance estimates, particularly reactance [86]. Finally, within-test variability necessitates rigorous quality-control procedures and repeated measurements, reinforcing the need for clearly standardized acceptability and repeatability criteria [86]. Accordingly, proposed numerical oscillometry thresholds should be interpreted as context-specific and hypothesis-generating, and should not be applied as universal diagnostic criteria until they have been robustly validated across platforms, protocols, and populations [98].

### 5.4. Multiple-Breath Washout (MBW)

MBW assesses ventilation distribution by measuring the clearance of an inert tracer gas during quiet tidal breathing and is regarded as highly sensitive to ventilation heterogeneity [99]. The principal global outcome is the LCI, which reflects the number of lung volume turnovers required to reduce the tracer-gas concentration to a predefined fraction of its starting value [99,100]. Higher LCI values indicate more uneven ventilation distribution [9]. Additional indices, including Scond and Sacin, have been developed to further characterize heterogeneity by attributing contributions preferentially to more conducting-airway-dominant (Scond) versus more distal/acinar regions (Sacin), although interpretation depends on the specific method and underlying modelling assumptions [101]. Mechanistically, MBW primarily captures ventilation heterogeneity arising from patchy small-airway narrowing and/or closure and the resulting regional variation in time constants [99].

LCI offers an effort-independent, sensitive marker of ventilation heterogeneity that may detect early or mild disease when spirometric indices, particularly FEV_1_, remain within the normal range, supporting its appeal for longitudinal monitoring and as a candidate endpoint in clinical trials [102]. In healthy participants, LCI varied with body size, particularly in early childhood; therefore, the study provided height-based reference equations and defined abnormality as an ULN at the 97.5th centile (z = 1.96; ≈predicted LCI + 1.18), while suggesting a practical fixed cutoff of LCI = 7.56 [103]. In pulmonary hypertension (PH), MBW has been reported to provide a practical means of detecting otherwise unrecognized small-airway involvement by demonstrating abnormal ventilation heterogeneity (e.g., elevated LCI and/or Sacin/Scond), discriminating PH from control groups and adding information beyond conventional spirometric flow measures, such as maximal expiratory flow (MEF) [104]. In a cohort of children aged 3–15 years, LCI, which reflects small-airway ventilation heterogeneity, remained abnormally elevated in a subset of symptom-controlled patients with prior severe asthma, with 27% exhibiting LCI above the healthy-control ULN, consistent with persistent SAD despite apparent clinical control [105].

From an operational standpoint, MBW requires only tidal breathing and is therefore less dependent on forced expiratory effort than spirometry [99]. Nonetheless, the technique can be time-consuming and is sensitive to leaks and breathing irregularities [99]. In addition, differences in tracer gas selection, device-specific algorithms, and analytical approaches can influence comparability across systems and studies, underscoring the need for methodological consistency when MBW is used for phenotyping or longitudinal assessment [99,106].

### 5.5. Fractional Exhaled Nitric Oxide (FeNO)

FeNO is a non-invasive biomarker of type 2 airway inflammation and is widely used for inflammatory phenotyping in asthma and related airway diseases [107]. Although FeNO is sometimes discussed in relation to distal airway involvement, particularly when measured across multiple expiratory flow rates to help differentiate proximal from more peripheral sources of NO production, it should not be construed as a direct measure of small-airway mechanics or ventilation heterogeneity [107]. Rather, FeNO provides complementary information on inflammatory activity that may contribute to mucosal oedema and airway narrowing, including within the small airways [108]. In this context, FeNO has been reported to reflect aspects of small-airway inflammation, and dynamic FeNO assessments (e.g., pre-/post-bronchodilator change, ΔFeNO) may offer SAD-relevant insight without directly quantifying dysfunction. For example, ΔFeNO has been shown to correlate with the oscillometry-derived small-airway index R5-R20, but not with conventional spirometric indices; moreover, its association with poorer asthma control appears to be mediated largely through SAD [109]. In addition, FeNO assessed at higher flow (e.g., FeNO350, measured at 350 mL/s) has been proposed as a prognostic inflammatory biomarker; higher baseline FeNO350 has been reported to predict clinical remission, supporting potential utility for monitoring distal airway inflammation and treatment response in selected settings [110].

Interpretation of FeNO requires attention to important confounders, as levels are influenced by atopy, cigarette smoking, respiratory infections, inhaled corticosteroid exposure, and testing conditions [111]. Moreover, the relationship between FeNO and physiological abnormalities compatible with SAD varies across diseases and phenotypes. FeNO should therefore be interpreted primarily as a complementary phenotyping biomarker of airway inflammation and potential corticosteroid responsiveness, rather than as a direct measurement of SAD or small-airway mechanics [108].

**Figure 2:**
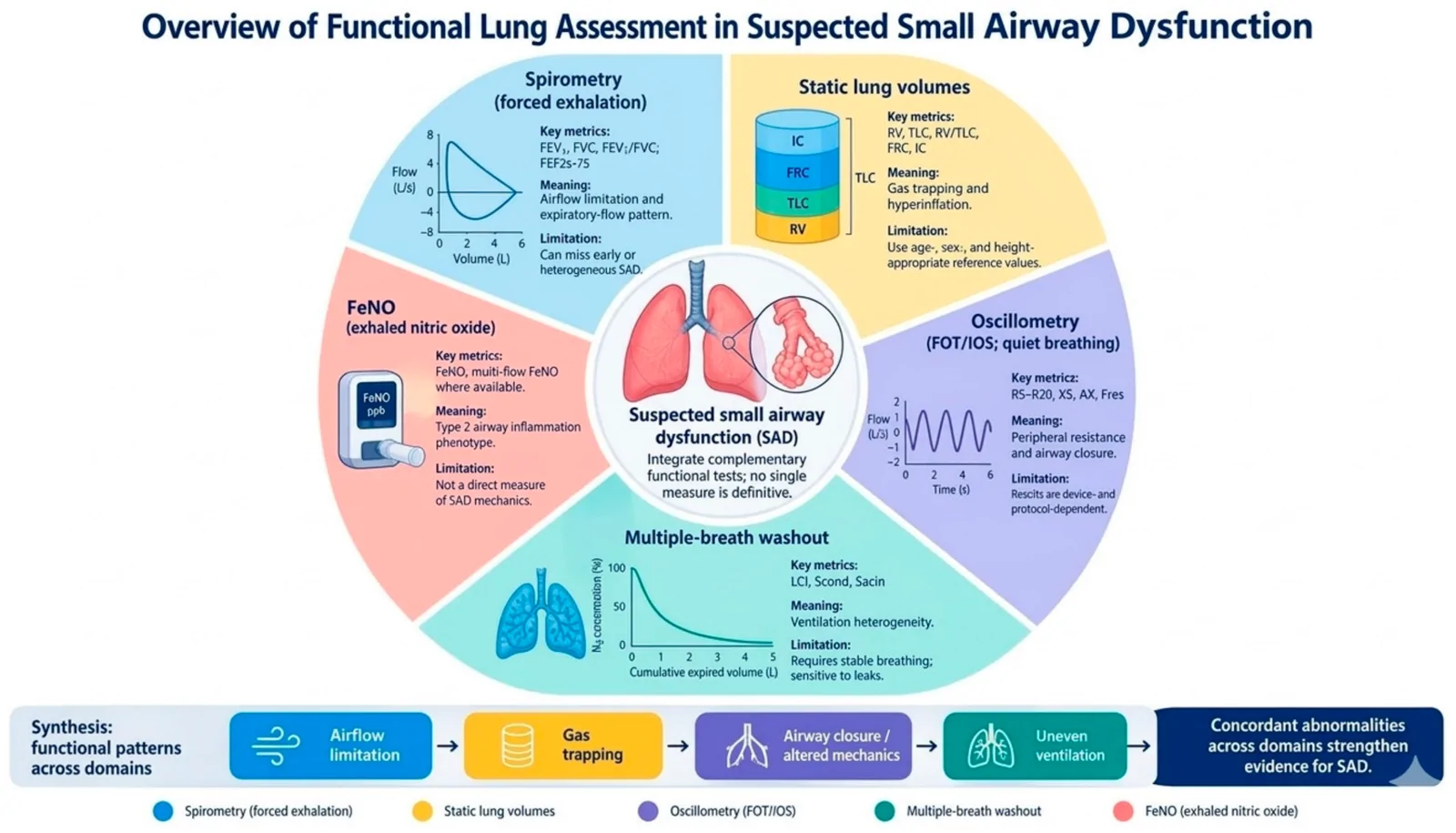
General approach to functional lung assessment and key parameters relevant to small airway evaluation.

To improve accessibility for readers, Figure 2 provides an overview of the general approach to functional lung assessment, summarizing the principal test modalities and key metrics used to characterize airflow, lung volumes, respiratory mechanics, ventilation distribution, and airway inflammation.

### 5.6. Emerging Technologies for the Detection and Phenotypic Classification of SAD

Emerging approaches, including artificial intelligence and machine-learning algorithms that analyze lung-function curve patterns and integrate multiple physiological parameters, may improve the early detection and phenotypic classification of SAD. For example, in symptomatic individuals with preserved pulmonary function, the combination of IOS and machine-learning algorithms substantially improved the diagnostic accuracy for SAD compared with individual oscillometric parameters alone [112]. In a large COPD gene cohort, deep-learning analysis of expiratory flow-volume curves identified computed tomography (CT)-defined functional small airway disease more accurately than conventional spirometric indices, demonstrating the potential of machine-learning approaches to detect SAD-related phenotypes and support targeted COPD management [113]. In parallel, advanced functional imaging techniques, such as quantitative high-resolution computed tomography (HRCT) and hyperpolarized gas magnetic resonance imaging (MRI), can provide regional assessment of air trapping, ventilation heterogeneity, and small-airway structural abnormalities. Across diverse populations, HRCT findings support an association between SAD and structural small-airway abnormalities, including airway-wall thickening, luminal narrowing, reduced bronchial counts, and radiographic features of small airway disease [114,115]. Additionally, Fluorine-19 MRI using inhaled perfluoropropane enabled regional quantification of ventilation defects and detected bronchodilator-responsive ventilation heterogeneity in asthma and COPD, supporting its potential value for assessing SAD beyond conventional spirometry [116]. Together, these multimodal approaches may complement conventional pulmonary function testing and support more precise characterization of SAD; however, further validation, standardization, and evaluation of clinical feasibility are required before their routine implementation.

However, the present review focuses on lung function-based assessment of SAD; emerging functional imaging techniques, artificial intelligence, and machine-learning algorithms represent important complementary approaches and warrant dedicated evaluation in future reviews.

The SAD mechanisms and their expected lung function signatures, including spirometry, static lung volumes, IOS/FOT, MBW, and FeNO, are summarized in Table 1.

## 6. Applications in Chronic Respiratory Diseases

### 6.1. Asthma

SAD is increasingly recognized as a relevant component of asthma across the spectrum of disease severity, including in individuals with preserved spirometry who nonetheless report persistent symptoms or exhibit poor disease control [38,39,117]. Distal airway inflammation, mucus plugging, and airway smooth muscle dysfunction can promote ventilation heterogeneity and expiratory airway closure, with these abnormalities often fluctuating in response to triggers, variable disease activity, and treatment adherence [26,118]. In this setting, oscillometry can identify abnormal peripheral airway mechanics, such as elevated R5-R20, and abnormal reactance-derived measures (e.g., Xrs and AX); in some contexts, it may be more sensitive than FEV_1_ for detecting treatment-related changes [119,120]. An international Delphi consensus has further concluded that respiratory oscillometry provides clinically useful information that complements spirometry in adults with asthma, assisting in the detection and grading of abnormal lung mechanics and in the assessment of bronchodilator responsiveness, thereby supporting its role in routine monitoring and interpretation [121]. Among patients with asthma with normal spirometry, increased peripheral airway resistance assessed by IOS has been associated with poorer asthma control and a greater burden of symptoms and exacerbations [122]. Similarly, another study reported that, among adults with asthma and preserved spirometry, higher R5-R20 values demonstrated strong discriminative performance for poorly controlled asthma [38].

MBW may detect ventilation heterogeneity even when conventional spirometric indices are normal, supporting the concept of “small-airway-predominant” asthma phenotypes [123]. FeNO can provide complementary information by reflecting airway inflammatory activity, particularly type 2 inflammation, although relationships with physiological indices of SAD may differ by phenotype and treatment exposure [107,110,124]. Overall, integrating spirometry with oscillometry and, where available, FeNO and/or MBW may improve the identification and phenotyping of distal airway involvement in asthma, particularly when abnormalities are not detected by spirometry alone. These measures may serve as adjunctive tools for clinical assessment and longitudinal monitoring; however, their role in guiding the selection or intensity of anti-inflammatory therapy and inhaler device choice remains investigational and requires further validation [45,110,123,124].

### 6.2. COPD

In COPD, abnormalities of the small airways together with parenchymal destruction act in concert to produce airflow limitation, gas trapping, and lung hyperinflation [4]. SAD is a major determinant of exertional dyspnea through the development of dynamic hyperinflation and reduced inspiratory reserve, pathophysiological effects that are often incompletely captured by FEV_1_ alone [1]. Although spirometry typically demonstrates persistent airflow obstruction, fluctuations in symptoms and functional limitation may correspond more closely to indices of hyperinflation than to changes in FEV_1_ [12]. Static lung volume measurements frequently demonstrate increased RV and RV/TLC, while IC is often reduced and may further decline dynamically during exercise [12]. Lung hyperinflation should be assessed using appropriate reference equations, with TLC, end-expiratory lung volume (EELV), RV, and RV/TLC interpreted relative to the ULN or z-scores where available. In COPD, elevated static or dynamic lung volumes may reflect airflow limitation and air trapping [77].

Oscillometry often reveals prominent reactance abnormalities consistent with airway closure and ventilation heterogeneity, supporting its utility as a sensitive marker of peripheral airway mechanics in COPD [121,125]. In addition, IOS has been suggested as a feasible diagnostic adjunct to spirometry. Key indices, including R5, Fres, AX, R5-R20, and X5, demonstrated good discriminatory performance, with increased airway resistance and abnormal reactance, particularly higher AX, indicating potential diagnostic value in settings where spirometry cannot be performed reliably [126].

MBW may show marked abnormalities consistent with extensive ventilation heterogeneity; however, feasibility in severe COPD may be constrained by test duration and ventilatory limitation [9,127]. Overall, in COPD, a physiological assessment strategy that prioritizes lung volumes (as direct measures of air trapping and hyperinflation) and is complemented by oscillometry may provide a more mechanistic understanding of dyspnea and facilitate monitoring of therapeutic effects that are not adequately reflected by spirometric indices alone [45,128].

Beyond its potential role in explaining symptom mechanisms, physiological abnormalities compatible with SAD in COPD may help identify patients at higher risk. This information may support closer follow-up and proactive management; however, prospective validation is needed to determine whether SAD-guided strategies can reduce exacerbations or slow disease progression [129]. Notably, in stable COPD, the addition of inhaled corticosteroids (ICSs) to Long-Acting Muscarinic Antagonist (LAMA)/Long-Acting Beta2-Agonist (LABA) therapy has been associated with a significant improvement in Fres despite no corresponding spirometric change, underscoring the potential value of IOS-based monitoring for detecting treatment responses at the level of small-airway mechanics [130].

### 6.3. Bronchiolitis

Bronchiolitis comprises a heterogeneous group of disorders characterized by inflammation and/or fibrosis of the distal airways, frequently resulting in disproportionate small-airway narrowing and an increased tendency toward airway closure [131]. Depending on the underlying subtype, the disease course may be progressive, and the physiological presentation may be variable, including mixed functional patterns [131]. In this context, oscillometry may be particularly informative for capturing abnormalities related to airway closure propensity and gas trapping [132]. For example, in pediatric bronchiolitis obliterans (BO), IOS measures, particularly R5 and AX, have been reported to identify physiological abnormalities compatible with SAD and to discriminate BO from controls with high diagnostic accuracy. These findings suggest that IOS may serve as a practical adjunct when conventional pulmonary function testing is difficult to perform [132].

Moreover, IOS indices, including Fres and R5-R20, have been shown to correlate more strongly than spirometric measures with structural airway changes, demonstrating negative associations with airway inner area and positive associations with airway wall area. These findings support the view that oscillometry may be more sensitive than spirometry for linking airway remodeling to physiological impairment in bronchiolitis [133]. While spirometry often demonstrates airflow obstruction, it may incompletely represent distal airway pathology when disease distribution is heterogeneous or patchy [132].

### 6.4. Bronchiectasis

In bronchiectasis, airway architectural distortion, mucus plugging, and infection-driven inflammation can generate marked regional heterogeneity in ventilation and contribute to peripheral airflow limitation, even when conventional spirometric obstruction is mild [134]. MBW may be particularly informative for quantifying ventilation heterogeneity attributable to mucus plugging and spatially heterogeneous disease involvement [135]. Oscillometry, performed during tidal breathing, may likewise provide useful information regarding peripheral airway mechanics [37]. In adults with non-cystic fibrosis bronchiectasis, IOS has been proposed as a valuable adjunct for evaluating physiological abnormalities compatible with SAD. It may identify such abnormalities that are not apparent on spirometry, particularly in earlier disease, and may complement standard physiological assessment. However, associations between IOS-defined SAD and clinically relevant outcomes, such as exacerbations, remain inconsistent, underscoring the need for standardized longitudinal studies to clarify its prognostic utility and optimal interpretation [136].

### 6.5. Cystic Fibrosis (CF)

Cystic fibrosis is characterized by early and progressive involvement of the small airways, driven by impaired mucociliary clearance, mucus plugging, recurrent infection, inflammation, and airway remodeling [137]. These pathological changes may produce regional ventilation heterogeneity before substantial abnormalities become evident on conventional spirometry [138]. MBW, particularly the LCI, is therefore highly relevant in CF because it is sensitive to early peripheral airway disease and may detect functional impairment in patients with preserved FEV_1_. In both clinical research and longitudinal monitoring, elevated LCI has been associated with structural lung disease, pulmonary exacerbations, and response to therapeutic interventions. In children aged 3–12 years with mild primary ciliary dyskinesia or CF, LCI derived from MBW detected abnormal lung function in approximately 58–59% of patients, substantially more than FEV_1_ (17–18%), supporting its value as a sensitive marker of early SAD and ventilation heterogeneity [102]. Although MBW requires specialized equipment, standardized procedures, and careful quality control, it provides a valuable complementary measure of small-airway involvement and ventilation heterogeneity in CF, especially in children and individuals with mild disease. In children with cystic fibrosis, IOS detected abnormal airway resistance and reactance compared with healthy controls and sensitively reflected deterioration during acute exacerbations and improvement after treatment, supporting its usefulness for pulmonary-function monitoring, particularly in younger children unable to perform reliable spirometry [139].

### 6.6. Interstitial Lung Diseases (ILDs) with Small-Airway Involvement

Although ILDs are classically characterized by restrictive physiology, concomitant small-airway inflammation and/or peribronchiolar fibrosis may occur and can contribute to symptoms, air trapping, and ventilation heterogeneity [140]. This consideration is particularly relevant in selected ILD subtypes and in smoking-related or hypersensitivity-related disorders in which bronchiolar involvement is prominent [140].

Importantly, restriction may obscure spirometric evidence of obstruction; the FEV_1_/FVC ratio is often preserved or even elevated despite clinically meaningful distal airway pathology [141]. Accordingly, oscillometry (IOS/FOT) may represent a useful adjunct for the detection and physiological characterization of SAD in patients with ILD [142]. For example, in rheumatoid arthritis, IOS indices consistent with SAD (including higher R5-R20, increased AX, more negative X5, and elevated Fres) were reported to be abnormal more frequently among patients with HRCT evidence of pulmonary involvement (including ILD) than was suggested by spirometry alone. Moreover, increased Fres was significantly associated with HRCT abnormalities, supporting the potential value of combining IOS with spirometry to enhance the non-invasive detection and monitoring of RA-related ILD and accompanying small-airway involvement [143]. Similarly, in mild pulmonary fibrosis without overt airflow obstruction, oscillometry—particularly measures of peripheral resistance, such as R5-R19—has been proposed as a complement to standard pulmonary function testing for identifying physiological abnormalities compatible with small-airway involvement and for flagging individuals with greater physiological and potentially prognostic risk [68]. In addition, in newly diagnosed idiopathic inflammatory myopathy-associated ILD, SAD has been suggested by abnormalities in lung-volume measurements, indicating that plethysmography-derived lung-volume assessment may contribute to the detection of early distal airway dysfunction in this population [144].

## 7. Practical Advantages, Limitations, and Common Interpretive Pitfalls

### 7.1. Spirometry

Spirometry is widely available, supported by standardized quality-control frameworks, and has strong diagnostic and prognostic utility [145]. However, it is relatively insensitive to early or patchy SAD and is effort-dependent; in particular, mid-expiratory flow measures (e.g., FEF25–75) show substantial variability and limited specificity [21,146]. Clinically, this means isolated reductions in FEF25–75 should not be overinterpreted, and persistent symptoms should not be dismissed simply because “spirometry is normal” without considering additional small-airway assessments (e.g., oscillometry, lung volumes, or imaging) [65].

### 7.2. FOT/IOS

Only tidal breathing is required, and oscillometry is sensitive to peripheral airway mechanics; it is therefore useful when spirometry is difficult or not feasible and can capture both resistance (e.g., R5, R5-R20) and closure-/heterogeneity-related reactance (e.g., X5, AX, Fres) [45]. However, interpretation can be limited by inter-device variability, the need for rigorous technique and quality control, and reference standards that are still evolving [96]. Common sources of error that can materially bias results include inadequate cheek support (upper-airway shunting), air leaks around the mouthpiece, irregular breathing or hyperventilation, swallowing/coughing, glottic/vocal-cord closure, poor posture or tongue position, and failure to remove artifacts; in addition, comparing values across different oscillometry systems without harmonization (different signal waveforms, frequency sets, and processing algorithms) can lead to misleading between-study or between-clinic comparisons [86,96].

### 7.3. MBW

MBW provides a relatively direct quantification of ventilation heterogeneity (e.g., via LCI) using only tidal breathing, which makes it feasible for patients who struggle with forced maneuvers [99]. However, MBW typically requires a longer test time and is technically demanding, with results highly sensitive to system leaks, poor seal/mouthpiece leaks, and inadequate equipment calibration/wash-in–wash-out stability [99]. In addition, comparability can be problematic across tracer gases, devices, and analysis software because differences in signal processing, alignment, and calculation algorithms may yield different LCI values [99]. Clinically, LCI should not be interpreted in isolation: breathing pattern (tidal volume and respiratory rate), assumptions about dead space, and strict test acceptability/quality criteria can materially influence results, and applying cross-device or cross-gas “universal” cutoffs may lead to misclassification unless device-specific reference values and thresholds are used [99].

### 7.4. Static Lung Volumes

Static lung volumes provide a direct physiologic readout of gas trapping (increasing in RV and RV/TLC) and hyperinflation (increasing in FRC and TLC), and these indices often correlate well with symptom burden, exercise limitation, and functional impairment in obstructive lung diseases [147]. Measuring lung volumes, however, requires specialized equipment and trained personnel, most commonly body plethysmography, and testing/interpretation can be more technically challenging in patients with severe obstruction (e.g., difficulty maintaining stable panting frequency/pressure, increased artifact risk) [147]. Key interpretive pitfalls include assuming that an elevated RV (or RV/TLC) reflects “small airways only”, when in fact it represents integrated respiratory mechanics (airway narrowing/closure plus contributions from lung elastic recoil and overall airway resistance), and failing to account for method-dependent differences: plethysmography may yield higher thoracic gas volume in obstructive disease compared with gas dilution/washout methods, so values should not be compared or trended across methods without acknowledging this bias [147].

### 7.5. FeNO

FeNO is a rapid, noninvasive biomarker of type 2/eosinophilic airway inflammation in appropriate clinical phenotypes and is most appropriately applied to inflammatory phenotyping, treatment selection, and longitudinal monitoring in those contexts (particularly in asthma) [148]. Importantly, FeNO does not directly quantify SAD or distal airway mechanics, and reported associations with physiological indices of airflow obstruction or ventilation heterogeneity are inconsistent and susceptible to confounding by atopy/allergic rhinitis, active smoking, recent respiratory infection, expiratory flow rate/measurement technique, and concurrent therapies, thereby limiting specificity for SAD [149]. A key interpretive pitfall is to treat FeNO as a surrogate of peripheral airway mechanics (e.g., inferring small-airway closure or increased distal resistance from FeNO values alone). Because FeNO can change substantially with anti-inflammatory treatment, most notably inhaled corticosteroids, adherence, and proximity to recent dosing-temporal “improvement” or “worsening” in FeNO should be interpreted primarily as a signal of inflammatory control rather than as evidence of meaningful change in distal airway mechanics [149].

The advantages, limitations, and interpretive pitfalls of spirometry, lung volumes, IOS/FOT, MBW, and FeNO are summarized in Table 2.

## 8. A Pragmatic Framework for Clinical Integration and Phenotypic Identification of SAD

A stepwise, question-driven framework can help clinicians and researchers select and integrate respiratory tests based on the following: (1) the specific clinical objective (screening vs. phenotyping vs. monitoring); (2) the feasibility and test quality (patient ability, time, equipment, operator expertise); (3) the dominant suspected mechanism (airflow limitation, small-airway closure/ventilation heterogeneity, gas trapping/hyperinflation, or inflammation), thereby improving interpretability and avoiding redundant or mismatched investigations.

*Step 1: Establish baseline physiology and diagnosis*

Obtain high-quality, American Thoracic Society (ATS) and European Respiratory Society (ERS) standard spirometry, adding bronchodilator testing when clinically indicated for the following [145]: (1) establishing the presence and severity of airflow obstruction (e.g., FEV_1_, FVC, FEV_1_/FVC); (2) characterizing bronchodilator responsiveness and its pattern across indices (e.g., changes in FEV_1_ and/or FVC suggesting improved flow vs. reduced air trapping); (3) interpreting flow-volume loop morphology (e.g., concavity, reduced peak flow, premature end-expiratory termination) to provide additional clues about airway mechanics and guide whether further small-airway-focused testing is warranted [145].

*Step 2: Determine whether symptoms are disproportionate or SAD is suspected despite spirometry*

Clinical “red flags” that should raise suspicion of small-airway involvement (and prompt consideration of additional testing) include dyspnea that seems disproportionate to the degree of FEV_1_ reduction, reduced exercise tolerance, nocturnal or highly variable day-to-day symptoms, recurrent exacerbations, and ongoing respiratory symptoms despite near-normal or only mildly abnormal spirometry [150]. However, small-airway involvement should be considered not only in patients with symptoms disproportionate to the degree of spirometric impairment, such as exertional dyspnea, reduced exercise tolerance, nocturnal or variable symptoms, recurrent exacerbations, or persistent respiratory complaints, but also in individuals with mild or apparently well-controlled asthma and preserved spirometry. In these patients, oscillometry may identify SAD not detected by conventional spirometry, which may be associated with impaired asthma control, exacerbations, and persistent airflow limitation [21,38].

*Step 3: Choose complementary SAD-focused tests based on the suspected abnormality*
  1. If gas trapping or hyperinflation is suspected, measure static lung volumes, including RV, RV/TLC, FRC, and inspiratory capacity (IC). Where available, also consider assessments that capture dynamic hyperinflation during exertion (e.g., serial IC measurements) to link physiology with exertional symptoms and activity limitation [151].
  2. If peripheral airway mechanics or airway-closure tendency is suspected, use FOT/IOS to evaluate frequency-dependent resistance (e.g., R5 and R5-R20) and reactance-domain indices (e.g., Xrs/X5, Fres, and AX) that reflect peripheral stiffness/heterogeneity and closure-related mechanics [152].
  3. If ventilation heterogeneity or very early disease is the main concern, consider MBW to quantify the global and compartment-specific ventilation heterogeneity using LCI—with optional indices, such as Scond and Sacin—when available to provide additional insight into conductive vs. acinar ventilation heterogeneity [135].
  4. If an inflammatory contribution is suspected (e.g., relevant asthma phenotypes), rather than replacing physiologic small-airway assessments, use FeNO as an adjunct biomarker to support inflammatory phenotyping and help guide/monitor anti-inflammatory therapy while recognizing it complements [124].
*Step 4: Use multidimensional endpoints for monitoring and treatment response*

Rather than relying on FEV_1_ alone, follow-up should incorporate patient-centered and clinically meaningful outcomes—including validated symptom scores, exacerbation frequency/severity, healthcare use, and functional capacity (e.g., exercise tolerance or activity limitation)—to better reflect real-world disease impact [153]. In parallel, physiological monitoring is most informative when the chosen endpoint matches the dominant pathophysiologic abnormality: for example, RV/TLC (or IC/FRC) when gas trapping/hyperinflation predominates, reactance-based oscillometry indices (Xrs/X5, AX ± Fres) when small-airway closure/heterogeneity is suspected, and LCI (±Scond/Sacin) when ventilation heterogeneity is the primary concern [74,84,99,147,154,155].

**Figure 3:**
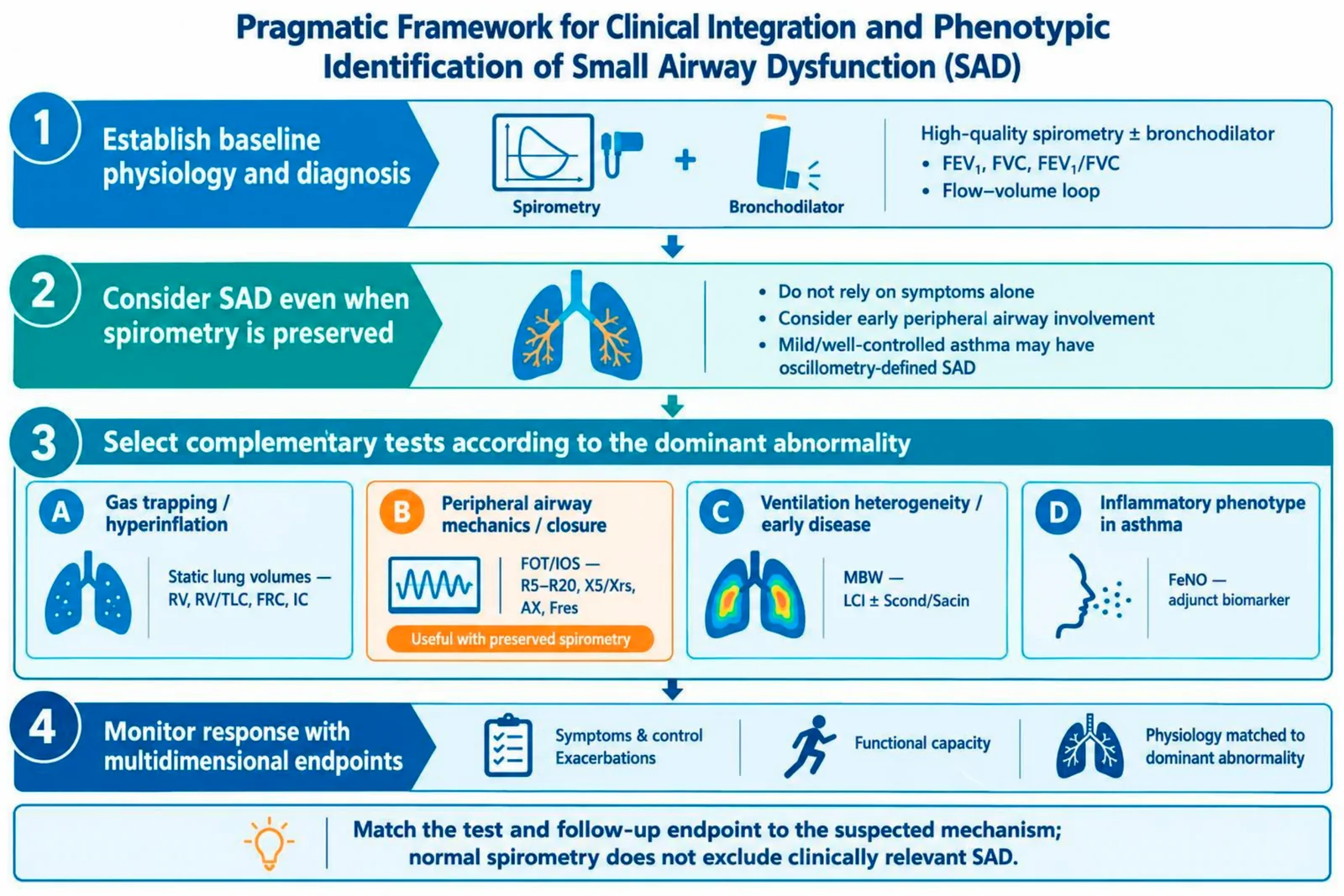
Proposed pragmatic framework for the assessment of small airway dysfunction in chronic respiratory diseases. Note: This figure represents an expert-informed, conceptual framework intended to support clinical assessment and research. It is not a validated clinical algorithm and should not be used as a standalone basis for diagnosis or treatment decisions.

A proposed pragmatic framework for clinical integration and phenotypic identification of SAD, using a stepwise selection and interpretation pathway, is shown in Figure 3.

## 9. Standardization Needs and Priorities for Future Studies

To support SAD-focused phenotyping and evaluate SAD-related physiological measures as potential clinical-trial endpoints, several methodological and evidence gaps must be addressed. Among these, two priorities are particularly important: establishing clinically meaningful thresholds and conducting longitudinal and interventional validation studies.

### 9.1. Harmonized Definitions and Standardized Measurement Procedures

Progress will require consensus on how physiological abnormalities compatible with SAD are defined across tests and diseases. This includes identifying which outputs from spirometry, lung volumes, oscillometry, and MBW should be interpreted together, while recognizing that the same numerical abnormality may have different implications in asthma, COPD, bronchiectasis, cystic fibrosis, bronchiolitis, or ILD. Standardized protocols and quality-control procedures are also essential, particularly for oscillometry and MBW. These should address patient preparation, posture, cheek support, leak and artifact handling, breathing stability, acceptability and repeatability criteria, reporting standards, and cross-device comparability [45,87,88,98].

### 9.2. Establishing Clinically Meaningful Thresholds and Responsiveness

A major priority is to establish disease-, population-, and device-specific reference ranges, diagnostic thresholds, and minimal important differences for key physiological measures, including R5-R20, X5/Xrs, AX, Fres, LCI, and, where appropriate, Scond and Sacin. These thresholds should be derived and validated within specific disease populations rather than applied universally across devices or conditions [156,157,158]. For example, prospective multicenter cohort studies could enroll sufficiently diverse and adequately powered populations across disease severity strata, with standardized testing at baseline and repeated assessments over at least 12–24 months. Candidate outcomes could include exacerbation frequency, symptom burden and symptom trajectories, health-related quality of life, exercise capacity, healthcare utilization, changes in conventional lung function, and imaging abnormalities. Analysis should adjust for major confounders, including smoking status, treatment exposure and adherence, comorbidities, and baseline obstruction or restriction severity [4,159,160]. Such studies could identify thresholds associated with clinically important outcomes and distinguish meaningful physiological changes from measurement variability or device-related effects.

### 9.3. Longitudinal and Interventional Validation

Longitudinal studies should determine whether physiological measures compatible with SAD predict or track clinically relevant outcomes, including exacerbations, hospitalization, functional limitation, and disease progression. Interventional trials should then evaluate whether treatments targeting relevant mechanisms, such as anti-inflammatory therapies, bronchodilators, airway-clearance interventions, antifibrotic agents, or phenotype-specific strategies, produce reproducible changes in SAD-related physiological measures and whether these changes are associated with patient-centered benefits [161,162,163].

For instance, randomized controlled trials could incorporate standardized oscillometry or MBW measures as key secondary endpoints, together with exacerbation rate, symptom scores, quality-of-life measures, and exercise outcomes. Follow-up should be adequate to capture both short-term physiological responses and longer-term clinical outcomes. These studies are needed before SAD-related physiological measures can be considered robust treatment-guiding tools or surrogate endpoints.

### 9.4. Mechanistic Linkage from Biology to Physiology

Finally, mechanistic studies should link biological processes, including epithelial injury, mucus dysfunction, inflammation, and airway remodeling, with physiological SAD-related readouts. Integrating physiological testing with imaging, biomarkers, and molecular profiling may clarify the biological meaning of individual endpoints and explain why their relationships with clinical outcomes differ across diseases and phenotypes [4,164,165,166].

## 10. Conclusions

SAD is a frequent, often early, and clinically relevant component of several chronic respiratory diseases. It reflects the interaction of multiple pathobiological processes, including epithelial injury and dysregulated repair, mucus retention or plugging, inflammation-related mucosal edema, altered smooth-muscle tone, and extracellular-matrix remodeling. Together, these processes may lead to small-airway narrowing, closure, and instability. Physiological abnormalities compatible with SAD include airway closure, ventilation heterogeneity, and gas trapping or hyperinflation, which may occur despite near-normal conventional spirometric indices and may not be fully captured by an FEV_1_-centered assessment alone.

In routine clinical practice, assessment should be pragmatic and guided by test availability and the clinical question. High-quality spirometry, including bronchodilator responsiveness when indicated, remains the appropriate first-line test to establish airflow limitation and evaluate reversibility. However, normal or near-normal spirometry does not exclude clinically relevant peripheral airway dysfunction, particularly in patients with asthma. Oscillometry is increasingly feasible in office-based settings because it requires minimal patient cooperation and can provide complementary information on peripheral airway mechanics and airway-closure-related abnormalities. Measures such as R5-R20, X5, AX, and Fres may support the identification of physiological abnormalities compatible with SAD, including in patients with preserved spirometry.

Static lung volumes may be useful when gas trapping or hyperinflation is suspected, whereas MBW and LCI measurements are more likely to be used in specialized centers or research settings to characterize ventilation heterogeneity and early peripheral airway dysfunction. The interpretation of all physiological measures should consider the disease context, patient population, device specifications, testing protocol, and quality-control standards, as these measurements provide indirect physiological evidence compatible with SAD rather than direct assessments of anatomical small-airway pathology.

Importantly, no universally accepted oscillometric cut-points currently define SAD across diseases, devices, or populations. Available thresholds for indices, such as R5-R20, X5, AX, and Fres, vary according to the device, reference equations, age, height, and disease population. Therefore, oscillometry results should be interpreted using device-specific reference values, z-scores or upper/lower limits of normal where available, longitudinal within-patient change, and concordance with clinical outcomes, rather than a single universal threshold. Before physiological markers of SAD can be routinely used for phenotypic stratification or treatment guidance, standardized definitions, validated disease- and device-specific thresholds, clinically meaningful changes, and prospective interventional evidence are required. Future research should prioritize harmonized testing and quality-control procedures, cross-device comparability, and longitudinal and interventional studies using clinically relevant SAD-related physiological and patient-centered outcomes.

## Figures and Tables

**Table 1:** SAD mechanisms and expected lung function signatures.

SAD Mechanism	Core Pathobiology	Expected Functional Abnormality Domains	Expected Lung Function Signatures
1. Epithelial injury and dysregulated repair	Recurrent epithelial damage with maladaptive repair and epithelial remodeling, contributing to reduced luminal caliber and altered mechanics	Small-airway narrowing; ventilation heterogeneity (often early, may precede clear spirometric impairment)	1. Spirometry: may be normal in early/patchy disease; may underestimate abnormalities prominent during tidal breathing 2. FOT/IOS: abnormalities in peripheral mechanics and closure-related indices (reactance and frequency-dependent resistance) 3. MBW: increased ventilation heterogeneity (e.g., higher LCI)
2. Mucus retention/plugging and impaired mucociliary clearance	Patchy mucus aggregates in small airways causing marked local luminal narrowing and regions of absent/poor ventilation; may sustain inflammation/infection cycles	Ventilation heterogeneity; airway closure propensity; gas trapping/hyperinflation	1. MBW: increased ventilation heterogeneity (abnormal washout indices, such as LCI) 2. Static lung volumes: gas trapping with higher RV and RV/TLC 3. FOT/IOS: peripheral airflow limitation patterns with abnormal resistance/reactance consistent with closure/heterogeneity
3. Inflammation-driven mucosal edema	Dynamic inflammatory swelling increases airway-wall thickness and narrows the lumen; may fluctuate over time and contribute to reversible components	Variable airflow limitation; airway closure; ventilation heterogeneity (often treatment-responsive)	1. Spirometry: may show bronchodilator responsiveness patterns (including changes suggesting reduced air trapping) 2. FOT/IOS: changes in resistance and reactance that track with activity/control and bronchodilator response 3. MBW: shifts in ventilation heterogeneity indices with disease activity 4. FeNO: adjunct marker of airway inflammation relevant to peripheral involvement (not a direct mechanics measure)
4. Smooth muscle dysfunction	Increased baseline tone and exaggerated bronchoconstriction; in asthma, distal airway narrowing is amplified by small baseline radii; in COPD, may coexist with reduced tethering forces	Bronchial hyperresponsiveness; partially reversible small-airway narrowing; may contribute to air trapping	1. Spirometry: may show change in FEV_1_; may also show improvements reflecting reduced air trapping (e.g., FVC and/or RV changes) that can be disproportionate to FEV_1_ change 2. FOT/IOS: improvement in resistance/reactance with bronchodilation/therapy, capturing distal mechanics 3. Static lung volumes: may improve if air trapping decreases
5. Matrix remodeling and loss of airway–parenchymal interdependence	Airway-wall fibrosis/stiffening and/or emphysematous loss of recoil reduces tethering, promoting fixed narrowing and expiratory collapse	Persistent low-volume flow limitation; airway closure tendency; gas trapping; ventilation heterogeneity (often less reversible)	1. Spirometry: reduced expiratory flow, especially at low lung volumes; may not localize to small airways 2. Static lung volumes: persistent gas trapping/hyperinflation (↑RV, ↑RV/TLC; possibly ↑FRC; ↓IC) 3. FOT/IOS: reactance abnormalities consistent with closure/heterogeneity; peripheral mechanics impairment 4. MBW: elevated indices consistent with ventilation heterogeneity (where feasible)

Abbreviations: SAD, small airway dysfunction; FOT, forced oscillation technique; IOS, impulse oscillometry; MBW, multiple-breath washout; LCI, lung clearance index; RV, residual volume; TLC, total lung capacity; FeNO, fractional exhaled nitric oxide; FEV_1_, forced expiratory volume in the first second; FVC, forced vital capacity; FRC, functional residual capacity; IC, inspiratory capacity. Note: The pathological mechanisms of SAD often overlap and interact in clinical practice; therefore, individual lung-function abnormalities should not be interpreted in isolation. Accurate assessment requires integration of multiple physiological parameters together with clinical context.

**Table 2:** Pros and cons and pitfalls for spirometry, lung volumes, IOS/FOT, MBW, and FeNO.

Modality	Key Advantages (Pros)	Key Limitations (Cons)	Common Interpretive Pitfalls/Sources of Error	Clinical Contexts, Considerations, and Recommendations
1. Spirometry	Widely available and supported by standardized quality-control frameworks, with strong diagnostic/prognostic utility in routine practice.	Relatively insensitive to early or patchy SAD and effort-dependent; mid-expiratory flow indices (e.g., FEF25–75) show substantial variability and limited specificity.	Overinterpreting isolated reductions in FEF25–75 as definitive SAD; dismissing persistent symptoms simply because “spirometry is normal” without considering additional small-airway-focused tests (e.g., oscillometry, lung volumes, imaging).	Recommended as first-line test in primary care and general clinical settings due to accessibility and ease of use. Useful for initial screening in all age groups but may be less sensitive in early disease or pediatric populations.
2. Static lung volumes (plethysmography and gas dilution)	Provide physiologic measures that may indicate gas trapping (↑RV, ↑RV/TLC) and hyperinflation (↑FRC, ↑TLC). Although these indices are clinically useful and may correlate with symptom burden, exercise limitation, and functional impairment in obstructive diseases, they reflect multiple aspects of respiratory mechanics and are not specific markers of SAD.	Require specialized equipment and trained personnel; testing/interpretation can be more technically challenging in severe obstruction (e.g., difficulty maintaining stable panting frequency/pressure, increased artifact risk).	Assuming elevated RV/RV: TLC reflects “small airways only” (it reflects integrated mechanics); comparing/trending values across methods without accounting for method-dependent differences (plethysmography may yield higher thoracic gas volume than gas dilution/washout in obstruction).	More suitable for tertiary or specialized centers with trained personnel. Recommended for detailed assessment in stable chronic respiratory disease and in older or severe patients where gas trapping is suspected.
3. IOS/FOT (oscillometry)	Requires only tidal breathing; sensitive to peripheral airway mechanics and can capture resistance metrics (e.g., R5, R5-R20) and closure-/heterogeneity-related reactance metrics (e.g., X5, AX, Fres).	Interpretation is limited by inter-device variability, the need for rigorous technique and quality control, and reference standards/cut-points that remain evolving.	Artefacts that bias results: inadequate cheek support (upper-airway shunting), mouthpiece leaks, irregular breathing/hyperventilation, swallowing/coughing, glottic/vocal-cord closure, poor posture/tongue position, failure to remove artifacts; comparing values across different oscillometry systems without harmonization (different waveforms/frequency sets/algorithms) can mislead.	Useful in pediatric and older adult populations because it requires minimal patient cooperation. It may be considered during both acute and stable phases to assess physiological abnormalities compatible with early SAD and to monitor treatment response, particularly in specialized clinics.
4. MBW (multiple-breath washout)	Relatively direct quantification of ventilation heterogeneity (e.g., LCI) using only tidal breathing; potentially sensitive to early small-airway disease.	Time-consuming and technically demanding; results highly sensitive to leaks and calibration/stability issues; comparability across tracer gases/devices/software can be problematic due to differing processing and algorithms.	Interpreting LCI in isolation; applying cross-device or cross-gas “universal” cutoffs (risk of misclassification) rather than device-specific reference values; not accounting for breathing pattern influences (tidal volume/respiratory rate), dead space assumptions, and strict acceptability/quality criteria.	Primarily used in research and specialized centers. May be considered in pediatric or early disease settings where detailed ventilation assessment is needed. Less practical in acute care due to time and technical demands.
5. FeNO	Rapid, noninvasive biomarker of type 2/eosinophilic airway inflammation in appropriate phenotypes; can support treatment decisions and monitoring in those settings.	Not a direct measure of SAD or distal airway mechanics; relationships with physiologic indices are variable and confounded by multiple factors (e.g., atopy/allergic rhinitis, smoking, recent infection, technique/flow, concurrent therapies).	FeNO should therefore be interpreted primarily as a complementary phenotyping biomarker of airway inflammation and potential corticosteroid responsiveness, rather than as a direct measurement of small-airway dysfunction or small-airway mechanics.	Useful as a complementary biomarker in both primary and specialized care to phenotype airway inflammation. Particularly helpful in guiding anti-inflammatory treatment in eosinophilic phenotypes across age groups and disease phases.

Abbreviations: SAD, small airway dysfunction; FEF25–75, forced expiratory flow at 25–75% of the FVC; RV, residual volume; FRC, functional residual capacity; TLC, total lung capacity; IOS, impulse oscillometry; FOT, forced oscillation technique; R5, resistance at 5 Hz; R5-R20, resistance at 5 Hz minus resistance at 20 Hz; X5, reactance at 5 Hz; AX, area of reactance; Fres, frequency resonance; MBW, multiple-breath washout; LCI, lung clearance index; FeNO, fractional exhaled nitric oxide.

## Data Availability

No new data were created or analyzed in this study. Data sharing is not applicable to this article.
